# A 28 nt long synthetic 5^′^UTR (synJ) as an enhancer of transgene expression in dicotyledonous plants

**DOI:** 10.1186/1472-6750-12-85

**Published:** 2012-11-10

**Authors:** Shaveta Kanoria, Pradeep Kumar Burma

**Affiliations:** 1 Department of Genetics, University of Delhi South Campus, Benito Juarez Road, New Delhi, 110021, India

**Keywords:** Synthetic 5^′^UTR, Transgene expression, 35S promoter, Ω leader, AMV leader

## Abstract

**Background:**

A high level of transgene expression is required, in several applications of transgenic technology. While use of strong promoters has been the main focus in such instances, 5^′^UTRs have also been shown to enhance transgene expression. Here, we present a 28 nt long synthetic 5^′^UTR (synJ), which enhances gene expression in tobacco and cotton.

**Results:**

The influence of synJ on transgene expression was studied in callus cultures of cotton and different tissues of transgenic tobacco plants. The study was based on comparing the expression of reporter gene *gus* and *gfp*, with and without synJ as its 5^′^UTR. Mutations in synJ were also analyzed to identify the region important for enhancement. synJ, enhances gene expression by 10 to 50 fold in tobacco and cotton depending upon the tissue studied. This finding is based on the experiments comparing the expression of *gus* gene, encoding the synJ as 5^′^UTR under the control of 35S promoter with expression cassettes based on vectors like pBI121 or pRT100. Further, the enhancement was in most cases equivalent to that observed with the viral leader sequences known to enhance translation like Ω and AMV. In case of transformed cotton callus as well as in the roots of tobacco transgenic plants, the up-regulation mediated by synJ was much higher than that observed in the presence of both Ω as well as AMV. The enhancement mediated by synJ was found to be at the post-transcriptional level. The study also demonstrates the importance of a 5^′^UTR in realizing the full potential of the promoter strength. synJ has been utilized to design four cloning vectors: pGEN01, pBGEN02, pBGEN02-*hpt* and pBGEN02-*ALS*^*dm*^ each of which can be used for cloning the desired transgene and achieving high level of expression in the resulting transgenic plants.

**Conclusions:**

synJ, a synthetic 5^′^UTR, can enhance transgene expression under a strong promoter like 35S as well as under a weak promoter like nos in dicotyledonous plants. synJ can be incorporated as the 5^′^UTR of transgenes, especially in cases where high levels of expression is required. A set of vectors has also been designed to facilitate this process.

## Background

Achieving high levels of transgene expression is one of the challenges for applications of transgenic technology. In order to achieve this, researchers have focused on using several viral and plant promoters like CaMV 35S, MMV, FMV and RbcS [1-3]. However, the expression of a gene is the outcome of the interplay of multiple processes including transcription, splicing, mRNA transport, mRNA stability, translation, protein stability and post translational modifications. Thus gene expression can be regulated not only by modulating transcriptional parameters but also by using post-transcriptional parameters like using optimal codons or by including 5^′^ and 3^′^ leader sequences. A lot of research has been focused on the transcriptional control mechanisms like testing various promoters [4-6], developing synthetic promoters [7,8] and using enhancer domains [9,10]. However, the importance of 5^′^UTRs on gene expression has not been explored to its fullest potential.

Most studies on 5^′^UTRs have focused on elucidating the importance of viral leader sequences [11-13]. For example, the 67 nt long 5^′^UTR derived from the Tobacco mosaic virus (TMV) RNA, widely called as ‘Ω leader’ has been shown to enhance the translation of foreign gene transcripts both *in vivo* and *in vitro*. Ω, is also one of the most well studied leader sequences shown to enhance reporter gene expression in tobacco mesophyll protoplasts, *Xenopus laevis* oocytes, *E. coli*[14], in rabbit reticulocytes, as well as in wheat germ extract [15]. Another viral leader sequence known to be a translational enhancer is that of the alfalfa mosaic virus RNA4. This leader when used as a 5^′^UTR downstream to 35S promoter enhances GUS activity by 8 fold in comparison to the pBI121 binary vector at a post-transcriptional level [16]. Apart from viral leader sequences, several plant 5^′^UTRs have also been seen to enhance transgene expression. For example, the 5^′^UTR of *NtADH*[17], *sps1*[18], *rbcL* and *atpB* genes [19] demonstrate this capacity. However these have not yet been commercially exploited for transgene expression in plants. A synthetic 5^′^UTR has also been tested in the binary vector pSTART [20]. In pSTART, a 78 nt long synthetic stretch of DNA, containing poly(CAA) region, was incorporated as a 5^′^UTR downstream to the 35S promoter, which showed improvement in transgene expression over that achieved by pBI121. The designing was based on motifs present in the Ω leader sequence. This seems to be the only available report, wherein a synthetic 5^′^UTR has been shown to enhance transgene expression.

In our study, we demonstrate the potential of a small (28 nt long) synthetic 5^′^UTR, hitherto called as synJ, to enhance transgene expression levels, which is at par or even higher than what could be achieved with the Ω and AMV leader sequences in transgenic tobacco lines and in callus cultures of cotton. synJ was designed as a multiple cloning site containing *EcoR*I, *Sna*I and *Nco*I restriction enzyme recognition sites for developing expression cassettes with the 35S promoter [7]. The enhancing activity of synJ was a serendipitous observation. The present study apart from analyzing the influence of synJ also demonstrates that the 5^′^UTRs are important for realizing the full potential of the promoter strength.

## Results

### Constructs and experimental outline

**Figure 1:**
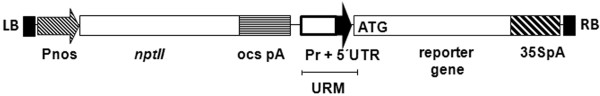
**Schematic representation of binary vectors (within T-DNA borders) developed for the transformation of tobacco and cotton.** The left and right borders of the T-DNA are designated as LB and RB, respectively. The selection marker gene (*nptII*) is driven by the nos promoter (Pnos) which also has a polyA signal of octopine synthase gene (ocspA). Different promoters (35S and nos) and reporter genes (*gus* and *gfp*) were used for creating various expression cassettes with a polyA signal of 35S (35SpA). A given combination of promoter and 5^′^UTR constituted an upstream regulatory module (URM).

Thirteen different constructs were developed in this study (Table 1). These constructs have different expression cassettes cloned into the binary vector pPZP200N [7]. Each expression cassette carries a promoter (either 35S or nos) driving the reporter gene β*-glucuronidase* (*gus*) or that encoding the green fluorescent protein (*gfp*) with a polyadenylation signal of the CaMV35S (35SpA). The reporter gene also encodes for a 5^′^UTR of the mRNA which is the main variable in different expression cassettes. The combination of the promoter and the 5^′^UTR has been called the ‘upstream regulatory module’ (URM). The different constructs are named after the URM they carry. As eleven of the thirteen constructs have *gus* as the reporter gene, all constructs named without an extension indicate that the reporter gene is *gus*. The general features of the binary construct are presented in Figure 1, while the sequences of different 5^′^UTRs are summarized in Table 2.

**Table 1:** List of constructs developed for the study

**S. No**	**Construct code**	**Promoter**	**gene**	**5'UTR**
1	35S(synJ)	35S	*gus*	synJ
2	35S(RT)	35S	*gus*	RT
3	35S(pBI)	35S	*gus*	pBI
4	35S (Ω)	35S	gus	Ω
5	35S(AMV)	35S	gus	AMV
6	nos(nos)	nos	*gus*	nos
7	nos(synJ)	nos	*gus*	synJ
8	nos(RT)	nos	*gus*	RT
9	35S(nos)	35S	*gus*	nos
10	35S(RT)-*gfp*	35S	*gfp*	RT
11	35S(synJ)-*gfp*	35S	*gfp*	synJ
12	35S(MsynJ)	35S	*gus*	MsynJ
13	35S(synM)	35S	*gus*	synM

**Table 2:** Sequence of the different 5′UTRs analyzed in the present study

**S. No**	**Name**	**Length (nt)**	**SEQUENCE (5′ to 3′)**
1	synJ	28	ACACGCTGGAATTCTAGTATACTAAACC**ATG**
2	RT	13	ACCTCGAGGGCCC**ATG**
3	pBI	37	GGGGGACTCTAGAGGATCCCCGGGTGGTCAGTCCCTT**ATG**
4	Omega (Ω)	77	ACCTCGAGTATTTTTACAACAATTACCAACAACAACAAACAACAAACAACATTACAATTACTATTTACAATTACACC**ATG**
5	AMV	45	ACCTCGAGTTTTTATTTTTAATTTTCTTTCAAATACTTCCATCCC**ATG**
6	MsynJ	28	ACAGGCGCTATCAATCCGAAGCTAAACC**ATG**
7	synM	28	ACACGCTGGAATTCTAGTATACTTTTCC**ATG**
8	nos	26	AGAGTCTCATATTCACTCTCAATCCC**ATG**

The region starts with the transcriptional start site (+1) and ends immediately upstream to the ATG (translational start site).

The influence of the different URMs on transgene expression was assessed initially in transformed cotton callus. Several independent callus were developed for each experiment. In order to reduce the influence of the physiological status of the callus on the expression levels, the transformed callus with expression cassettes that were to be compared were developed and analyzed simultaneously. In several cases, the observation made in the cotton callus was also confirmed in different tissues of T_0_ tobacco transgenic lines.

### Experiments on cotton callus

**Figure 2:**
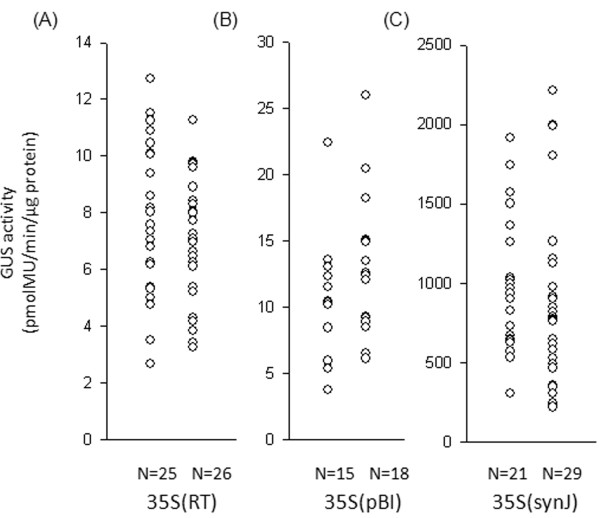
**GUS activity observed in cotton callus transformed with (A) 35S(RT), (B) 35S(pBI) and (C) 35S(synJ).** GUS activity was studied after 40–45 days of transformation. GUS activity recorded in callus transformed with 35S(synJ) was many fold higher than 35S(RT) and 35S(pBI) (see scales of Y-axis). Each circle represents the absolute GUS activity (pmolMU/min/μg protein) measured in one independent callus. GUS activity in this as well as in all the following figures is a mean of two independent reactions carried out for two time points. N in this figure and in later figures denotes the number of callus analyzed in each case.

In order to assess the influence of synJ on transgene expression, GUS activity was analyzed in several independent callus transformed with 35S(synJ) and compared to that of 35S(RT) and 35S(pBI) in two independent transformation experiments (Figure 2). In 35S(RT) and 35S(synJ), the *gus* gene is under the control of 35S (−343 bp) promoter from the CM1841 strain of CaMV, which has been used in plant expression vectors of the pRT series [21], whereas 35S(pBI) carries the full length 35S promoter (up to −835), as present in the binary vector pBI121. The GUS activity in case of 35S(RT) and 35S(pBI) was in a similar range i.e. 2.7 to 25.9 pmolMU/min/μg protein; whereas in case of callus transformed with 35S(synJ), it was found to be much higher (ranging between 222.5 to 2216.3 pmolMU/min/μg protein). In case of 35S(pBI), the *gus* expression cassette from the binary vector pBI121 was cloned into the vector pPZP200N as in the other two constructs. Thus, GUS activity, in the callus transformed with the original binary vector pBI121 was also recorded. The GUS activity observed ranged from 4 to 26 pmolMU/min/μg protein which was similar to that of 35S(pBI). Therefore, expression of the *gus* gene in the construct 35S(synJ) was found to be ~80 fold higher, based on mean values of GUS activity (pmolMU/min/μg protein) viz., 7.4 ± 0.35 for 35S(RT), 11.8 ± 0.86 for 35S(pBI) and 920.0±69.5 for 35S(synJ). This indicated a strong positive influence of synJ on the expression of the *gus* gene.

**Figure 3:**
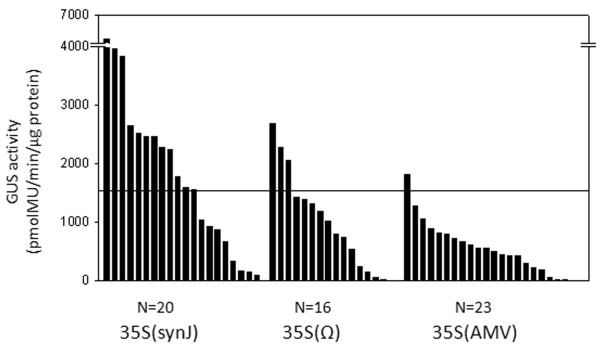
**GUS activity observed in cotton callus transformed with the constructs 35S(synJ), 35S(Ω) and 35S(AMV).** Each bar represents the mean GUS activity of the two experimental replicates measured with one independent callus. N denotes the number of callus analyzed in each case. The line represents the GUS activity of 1500 pmolMU/min/μg protein.

Further, we compared the influence of synJ with two other widely used 5^′^UTRs, Ω (from TMV) and the AMV leader, known to be translational enhancers [12,13]. The GUS activity (Figure 3) recorded in the callus transformed with the constructs 35S(Ω) (ranging from 4.6 to 2695.0 pmolMU/min/μg protein) as well as 35S(AMV) (ranging from 2.9 to 1814.6 pmolMU/min/μg protein) was lower than that of the callus transformed with 35S(synJ) (ranging from 98.9 to 6490.4 pmolMU/min/μg protein) (Figure 3). Sixty percent of the callus transformed with 35S(synJ) showed GUS activity higher than 1500 pmolMU/min/μg protein, whereas in case of the callus transformed with the constructs 35S(Ω) and 35S(AMV) most of the callus [80% in case of 35S(Ω) and 90% in case of 35S(AMV)] showed GUS activity lower than 1500 pmolMU/min/μg protein.

**Figure 4:**
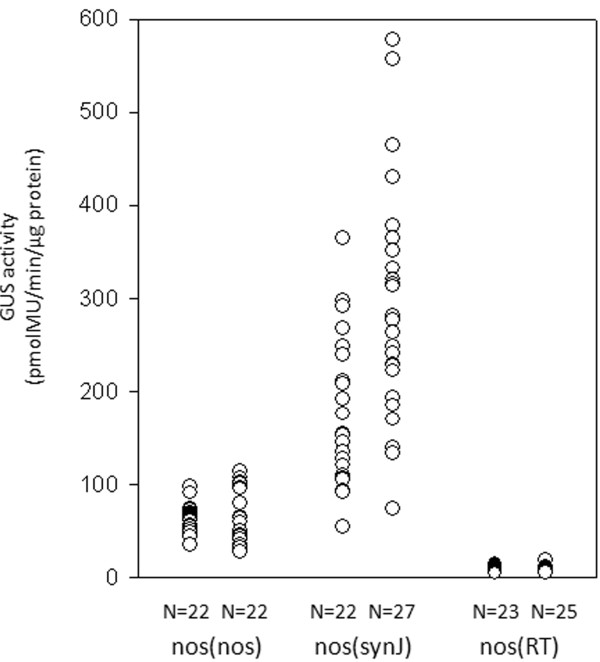
**Comparative analysis of nos(nos), nos(synJ) and nos(RT) constructs.** GUS activity in transformed cotton callus containing the expression constructs nos(nos), nos(synJ) and nos(RT) as observed in two independent experiments. Each circle represents GUS activity measured in one independent callus.

To test whether the enhancing activity of synJ could also be observed in conjunction with another promoter, we developed a new set of constructs with the promoter of the *nopaline synthase (nos)* gene derived from *Agrobacterium tumefaciens.* The promoter of the *nos* gene has been shown to have a 10 fold lower strength than that of the 35S promoter [22]. A set of plant expression constructs nos(nos), nos(synJ) and nos(RT) were therefore developed and two independent transformation experiments were carried out. The GUS activity observed in the callus transformed with nos(RT) construct was less than 20 pmolMU/min/μg protein, which was the lower limit of GUS activity observed in case of both nos(nos) and nos(synJ) transformed callus. Further, ~90% of the callus transformed with nos(synJ), showed GUS activity higher than 100 pmolMU/min/μg protein, which was the upper limit of GUS activity for nos(nos) transformed callus (Figure 4). These observations indicated that the presence of the 5^′^UTR nos, leads to higher expression than RT, but lower than what could be achieved with synJ.

**Figure 5:**
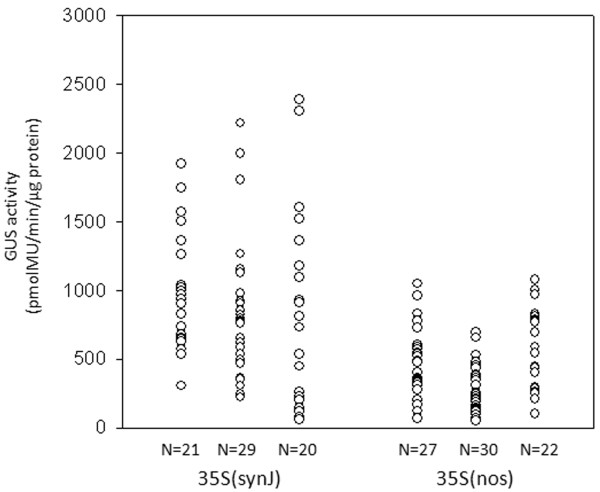
**Comparison of GUS activity driven by 35S(synJ) and 35S(nos).** GUS activity as observed in cotton callus when transformed with the constructs 35 S(synJ) and 35 S(nos) in three independent transformation experiments. Each circle represents GUS activity measured in one independent callus.

We further tested if the 5^′^UTR of nos functions similarly under the 35S promoter and compared the influence of 5^′^UTRs synJ and nos on *gus* gene expression when controlled by the 35S promoter. The constructs 35S(synJ) and 35S(nos) were used to develop transformed cotton callus in three independent experiments, the results of which are presented in Figure 5. The presence of nos 5^′^UTR resulted in a marked improvement in *gus* expression as compared to the constructs carrying either RT or pBI as 5^′^UTRs (Figure 2). However, the expression observed with 35S(synJ) was even higher wherein 30-40% of the callus showed GUS activity more than 1077 pmolMU/min/μg protein [the upper limit of GUS activity driven by 35S(nos)].

In order to study the influence of synJ on the expression levels of another transgene (*gfp*)*,* we developed two constructs 35S(RT)-*gfp* and 35S(synJ)-*gfp*. GFP fluorescence was recorded using 50 μg of total protein. No GFP fluorescence could be recorded in case of the callus transformed with 35S(RT); while fluorescence in the callus transformed with 35S(synJ)-*gfp*, ranged from 133.75 to 2456.5 F.U/100 μg of total protein.

### Analysis of transgenic tobacco plants

In order to substantiate the results obtained from the cotton callus, we analyzed some of the constructs in tobacco transgenic plants. We developed a number of independent transgenic lines with the constructs 35S(RT), 35S(pBI), 35S(synJ), 35S(Ω) and 35S(AMV).

**Figure 6:**
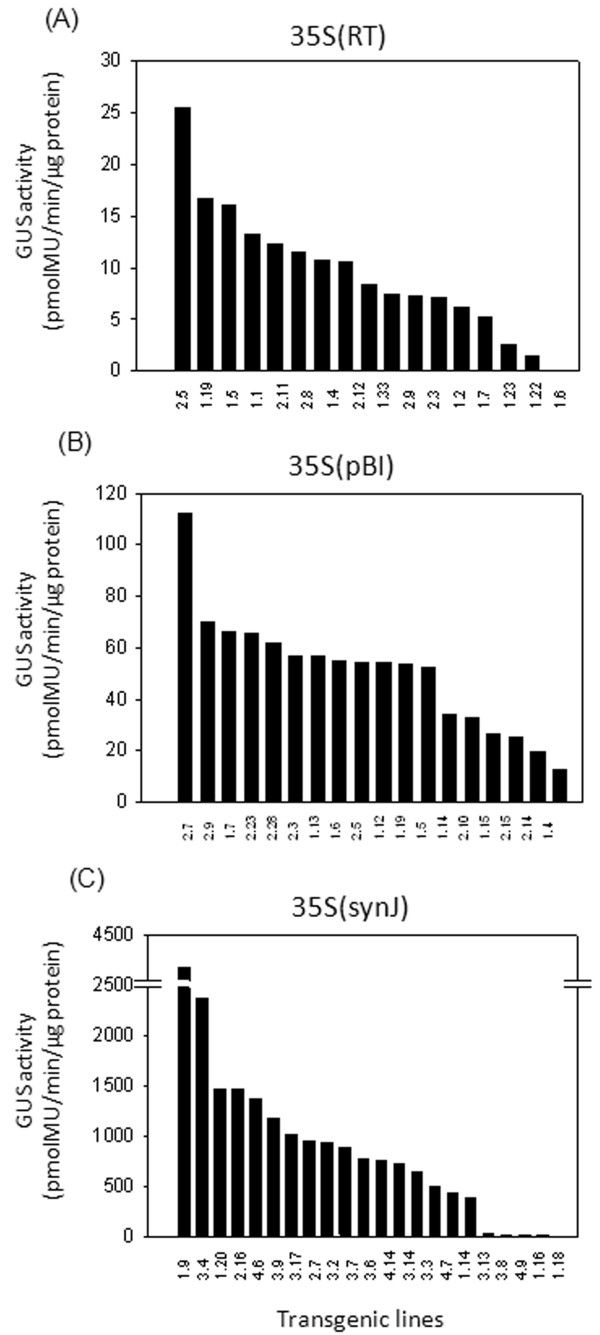
**Comparative analysis of 35S(RT), 35S(pBI) and 35S(synJ) constructs in the leaves of tobacco transgenic plants.** GUS activity has been represented as measured in the leaves of tobacco transgenic plants transformed with the constructs (**A**) 35S(RT), (**B**) 35S(pBI) and (**C**) 35S(synJ). The level of GUS activity with 35S(synJ) was much higher than the other two constructs. The scale of the Y-axis (i.e. GUS activity pmolMU/min/μg protein) is different for each graph.

**Figure 7:**
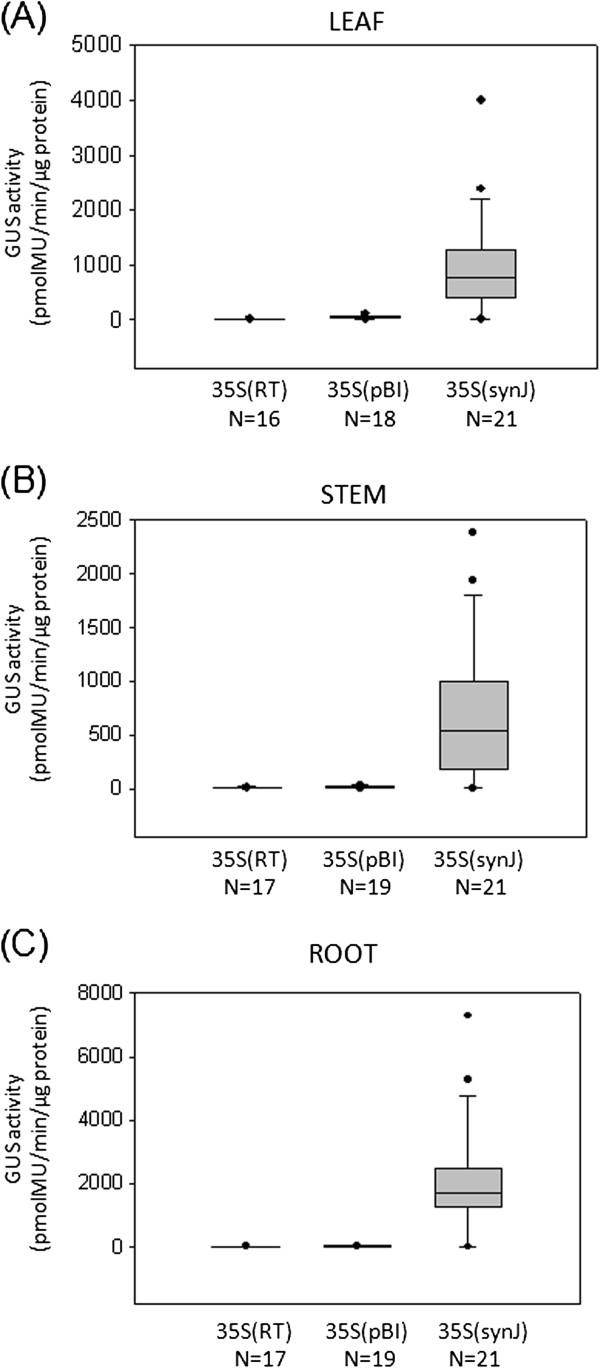
**Comparison of 35S(RT), 35S(pBI) and 35S(synJ) in the leaf, stem and root tissues of tobacco transgenic plants as a Box and Whisker plot.** The horizontal lines in the Box and Whisker plot represent 10, 25, 50, 75, and 90 percentiles. Extreme values are depicted as circles at the top and bottom of the plot.

The GUS activity recorded in the leaves of independent transgenic plants transformed with 35S(RT), 35S(pBI) and 35S(synJ) has been represented in Figure 6, while the distribution of GUS activity observed in leaf, stem and root tissues of these plants have been summarized as a ‘Box and Whisker plot’ in Figure 7. In each of these tissues, the GUS activity observed in plants transformed with 35S(RT) and 35S(pBI) was significantly lower than that with 35S(synJ).

**Figure 8:**
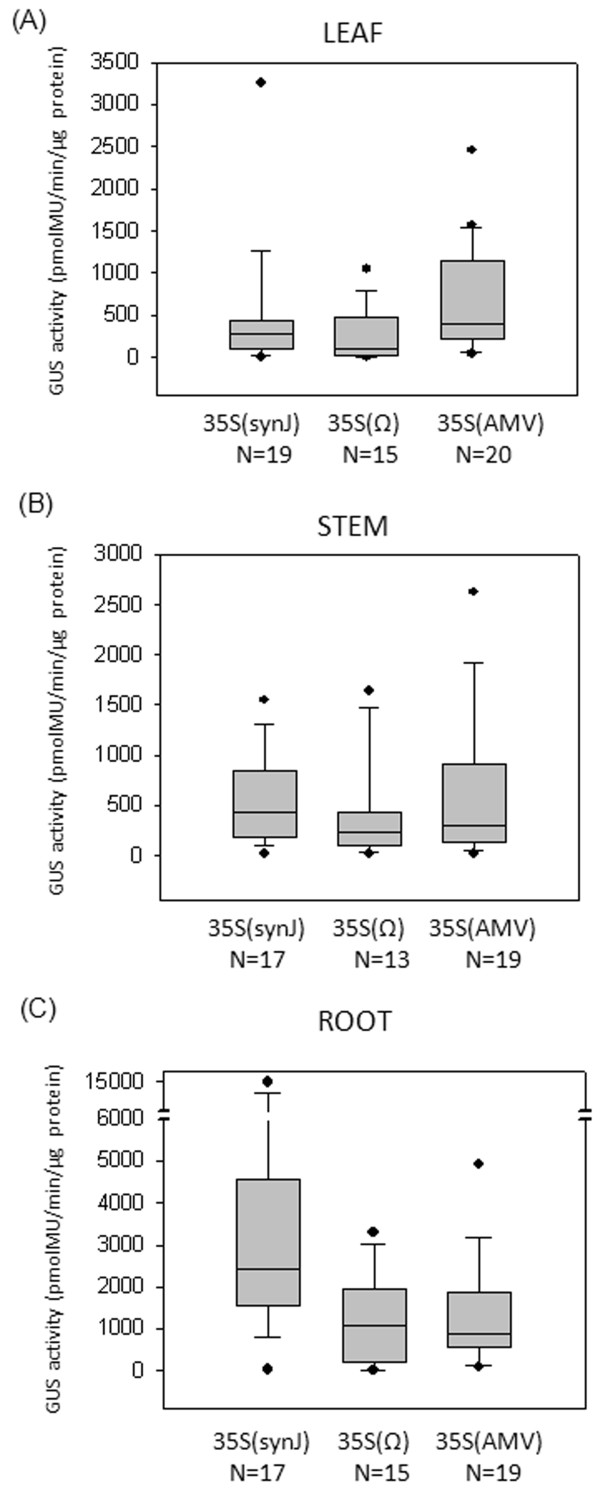
**Comparison of GUS activity driven by 35S(synJ), 35S(Ω) and 35S(AMV).** GUS activity measured in the (**A**) leaf (**B**) stem and (**C**) root tissues of tobacco transgenic lines transformed with the three constructs 35S(synJ), 35S(Ω) and 35S(AMV) presented as a Box and Whisker plot.

Further, the GUS activity in different tissues of transgenic lines transformed with 35S(synJ) was compared to that with lines transformed with 35S(Ω) and 35S(AMV), as represented in Figure 8. The influence of the three 5^′^UTRs i.e. synJ, Ω and AMV, on *gus* expression varied in different tissues. In the case of the leaf (Figure 8A), GUS activity in 62% of the lines transformed with 35S(AMV) was higher than 300 pmolMU/min/μg protein, while 47% and 27% of the lines transformed with 35S(synJ) and 35S(Ω) showed the same. In the case of the stem (Figure 8B), it was found that in contrast to 35S(synJ) wherein almost 47% of the lines showed GUS activity higher than 500 pmolMU/min/μg protein, only 15% and 30% of the lines transformed with 35S(Ω) and 35S(AMV) respectively showed *gus* expression in that range. Also, in the case of the roots (Figure 8C), 94% of the lines transformed with 35S(synJ) showed >1000 pmolMU/min/μg protein, while only 53% and 42% of the transgenic lines transformed with 35S(Ω) and 35S(AMV) respectively, showed GUS activity higher than 1000 pmolMU/min/μg protein.

### Modification of synJ and its influence on transgene expression

The 28 nt long synJ (ACACGCTGGAATTCTAGTATACT**AAACC**aug**G**) carries part of the near perfect translation initiation context (shown in bold) and an upstream region of 23 nt. A consensus TIC for dicots [23] has a sequence of aaaaaaaAA/CaAUGGC, wherein the initial A bases are less conserved. In order to analyze the role of these two regions and their influence on transgene expression, two mutant 5^′^UTRs were developed (Table 2). In synM, 3 bases (AAA) upstream to the CCATG were mutated to TTT, in order to break the consensus of TIC, while in MsynJ, 21 nt upstream to the TIC was changed. The modified 5^′^UTRs were cloned as a part of the *gus* transcript under the control of 35S promoter. The expression of the *gus* gene in these constructs was analyzed in transformed callus of cotton (Table 3). Forty five percent of the callus transformed with 35S(synJ) was observed to show GUS activity > 2000 pmolMU/min/μg protein.

**Table 3:** GUS activity observed in cotton callus transformed with constructs carrying synJ and its modified versions MsynJ and synM, downstream to 35S promoter

**Construct code**	**No. of calli analyzed**		**GUS activity (pmolMU/min/μg protein)**			
		**Range**	**Percentage of callus showing activity in the range of**			
			**< 500**	**500-1000**	**1000-2000**	**> 2000**
35S(synJ)	20	98.9-6490.4	20.0	15.0	20.0	45.0
35S(MsynJ)	22	3.2-1278.1	50.0	36.4	13.6	0
35S(synM)	14	5.2-802.4	78.6	21.4	0	0

Expression in callus transformed with 35S(MsynJ) was slightly higher than that obtained with 35S(synM), which was, however, much lower than that of 35S(synJ). Amongst the 22 callus transformed with 35S(MsynJ) ~13.6% showed GUS activity ranging between 1000 to 2000 pmolMU/min/μg protein whereas 50% of the callus showed GUS activity lower than 500 pmolMU/min/μg protein. On the other hand, 78.6% of the callus transformed with 35S(synM), showed GUS activity in the lower range.

To strengthen the above observations, the role of the two regions in synJ was also analyzed in transgenic tobacco plants developed with the expression constructs 35S(MsynJ), 35S(synM) and 35S(synJ). The *gus* expression levels were recorded in different tissues of the transformed lines. The influence of the given three 5^′^UTRs (synJ, MsynJ and synM) on *gus* expression was tested in the leaves of transgenic lines, the results of which have been presented in Table 4. In case of the leaf, GUS activity in ~33% of the lines transformed with 35S(synJ) was higher than 1000 pmolMU/min/μg protein, while none of the transgenic lines developed with 35S(MsynJ) and 35S(synM), had GUS activity in this range. In case of the stem, it was found that in contrast to 35S(synJ) wherein almost 62% of the lines showed GUS activity higher than 500 pmolMU/min/μg protein, only 25% of the lines transformed with 35S(MsynJ) and none transformed with 35S(synM) showed *gus* expression > 500 pmolMU/min/μg protein. Also, in case of the root, 81% of the lines transformed with 35S(synJ) showed GUS activity higher than 1000 pmolMU/min/μg protein, whereas none of the transgenic lines developed either with 35S(MsynJ) or 35S(synM) showed GUS activity in this range.

**Table 4:** Comparative GUS activity recorded in different tissues of transgenic tobacco lines transformed with constructs 35S(synJ), 35S(MsynJ) and 35S(synM)

**Tissue analyzed**	**GUS activity (pmolMU/min/μg protein)**	**35S(synJ)**	**35S(MsynJ)**	**35S(synM)**
Leaf	Range	15.9 - 3999.8	48.5 - 888.1	15.7 - 805.2
	No. of lines tested	21	18	22
	% Lines showing GUS activity >1000	33.3	0	0
	Mean ± S.E	951.3 ± 198.8	363.4 ± 54.6	329.9 ± 51.4
Stem	Range	3.3 - 2380.0	4.6 - 739.4	2.9 - 482.5
	No. of lines tested	21	20	25
	% Lines showing GUS activity >500	61.9	25.0	0
	Mean ± S.E	673.3 ± 136.8	321.3 ± 49.9	140.1 ± 27.5
Root	Range	4.4 - 7297.0	3.3 – 975.0	6.2 - 734.2
	No. of lines tested	21	19	24
	% Lines showing GUS activity >1000	81.0	0	0
	Mean ± S.E	1914.9 ± 371.9	424.4 ± 75.3	297.4 ± 45.9

### synJ enhances gene expression at a post transcriptional level

**Figure 9:**
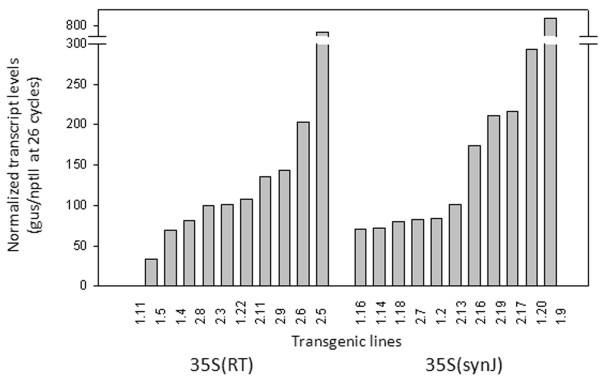
**Comparison of transcript levels driven by 35S(RT) and 35S(synJ).** Normalized levels of *gus* transcripts as observed in individual transgenic lines of tobacco developed with constructs 35S(RT) and 35S(synJ) carrying different 5^′^UTRs: RT and synJ. There was no major difference in the range of transcript levels observed between transgenics developed with 35S(RT) and 35S(synJ).

In order to check whether synJ exerted its influence at the transcriptional or post-transcriptional level, steady state levels of *gus* transcripts were measured by semi-quantitative RT-PCR of the RNA obtained from the leaves of T_0_ transgenic lines developed with the constructs 35S(RT) and 35S(synJ). The level of *gus* transcripts was measured in each case after 26 cycles of PCR amplifications, which was observed to be in the exponential phase. The level of *nptII* transcript was also measured to normalize variations. No major difference in the intensity of transcript levels which could account for the observed differences in GUS activity was seen between transgenic lines developed with the two constructs 35S(RT) and 35S(synJ) (Figure 9), suggesting that synJ enhances expression post-transcriptionally.

## Discussion

5^′^UTRs are known to influence gene expression [13,15,18,24,25]. When the coding region of a gene is cloned downstream to the transcriptional start site of the promoter in different cloning vectors, a 5^′^UTR is inadvertently introduced, until and unless the 5^′^UTR region of the transgene is an integral part of the expression cassette. Such stretches exist in commercially available vectors like pBI121 [26] which has been extensively used for comparing the strength of different promoters to that of the 35S [3,9,27-31]. Vectors like that of the pRT series [21] are also used to incorporate transgenes downstream to 35S for developing transgenic plants. In developing such cassettes, different 5^′^UTRs are introduced which can vary in length as well as sequence, depending on the particular vector and restriction enzyme(s) used.

In the present study, we observed that the presence of the synthetic 5^′^UTR, synJ led to several fold increase in expression of reporter genes *gus* and *gfp*. The enhancement is observed when the expression is under the control of a strong viral promoter like the 35S as well as under a promoter like nos, which is reported to be ~30 fold weaker than the 35S [32]. The level of enhancement was variable among the different tissues analyzed, the maximum observed in roots, followed by callus, stems and leaves in case of tobacco transgenic plants. Enhancement in cotton callus was similar to that observed in the case of the roots of transgenic tobacco. This enhancement is also evident when the present results were compared with the GUS activities under 35S promoter recorded in previous studies (Table 5).

**Table 5:** Examples of GUS activity driven by 35S promoter observed in different tissues of tobacco plants as reported in different studies

**Reference**	***Gus *expression studied in**	**Range of gus activity (pmol MU/min/μg protein)**
Sawant *et al.* (2001)*	Tobacco leaves	~0.13
De Amicis *et al.* (2007)*	Tobacco leaves	0.02-0.5
Outchkourov *et al.* (2003)*	Tobacco leaves	~31.7
Benfey *et al.* (1989)*	Tobacco transgenics (seedlings)	39.6-63.8
Jefferson *et al.* (1987)*	Tobacco leaves, stem & roots	0.2-0.6
Xu *et al.* (2010)*	Tobacco leaves	2.0-4.0
Sanger (1990)*	Tobacco leaves	~9.03
Present study	Tobacco leaves	2.56-3257.0
	Tobacco stems	16.0-1547.0
	Tobacco roots	23.3-14955.0

*In these papers GUS activity is expressed in different units. The value has been converted to pmolMU/min/µg protein for comparing it with the results of the present study.

The most widely used 5^′^UTRs for enhancing transgene expression in dicotyledonous plants are the leader sequences of the TMV (Ω leader) [13,17,25] and AMV [33-36]. Therefore, in the present study, we compared the influence of synJ to that of AMV and Ω leader sequences, which showed that the expression levels under 35S promoter observed in the leaves of transgenic tobacco plants with synJ as a 5^′^UTR, is equivalent to that observed with Ω and lower than that of the AMV leader sequence. In case of the stem, the synJ is equivalent to AMV and stronger than Ω. However, in case of the roots of transgenic tobacco plants as well as in transformed cotton callus, synJ showed much higher GUS activity in comparison to both Ω and AMV.

synJ, probably influences transgene expression at the post-transcriptional level as our results with RT-PCR did not show any difference in transcript levels between the constructs with and without synJ, which could account for the observed differences in GUS activity. The widely known 5^′^UTRs of the TMV and AMV have also been shown to give high levels of transgene expression by improving the translation of the mRNA. The Ω leader sequence carries a central poly(CAA) stretch which has been shown to be critical for the associated enhancement in gene expression [11,12]. Further, it has been demonstrated that the HSP101 binds to this poly(CAA) region and mediates binding of initiation factors eIF3 and eIF4G, thereby promoting translation initiation. However, based on our RNA-EMSA experiments, we did not observe any protein binding to the synJ (data not shown).

synJ carries a near consensus sequence for translation initiation context (TIC) and an upstream region. A consensus TIC ‘TAAACA**AUG**GCT’ was proposed by Joshi [37] based on an analysis of 79 different plant genes and a consensus sequence ‘AAAA/CA**AUG**GC’ in a later study [23] by analyzing 3643 plant genes. In the present study the TIC in synJ was TAAAC**C**AUGG. This does not carry a conserved A at −1 (considering ‘A’ of the AUG to be +1 base). Papers on the functional significance of TIC in plants, have given contradictory findings. While studies by Luehrsen and Walbot (1994) [38] and Lutcke *et al.*[39] showed that the AUG context was not very important in plants, reports by Guerineau *et al.*[40] and Lukaszewicz *et al.*[41] demonstrated improvement in the levels of transgene expression but only by 3 to 4 fold. Further, of the ~300 papers, which have cited Joshi′s work, wherein the consensus sequence for translation initiation was proposed [23,37] only a few papers have addressed the question of quantifying the influence of such a consensus sequence on the levels of gene expression. It seems that work to understand the role of a consensus TIC has been limited in the case of plants, as compared to studies carried out on the importance of the Kozak sequence in animal systems (reviewed in [42]). Studies by Lutcke *et al.*[39] and Luehrsen and Walbot [38] suggested that an optimal TIC might play a less significant role in plants than in animals. While Luehrsen & Walbot [38] used the maize transient assay system to carry out their study, Lutcke *et al.*[39] compared the *in vitro* translation efficiency between the reticulocyte lysate and the wheat germ system, wherein enhancement in transgene expression was observed in the former case but not in the latter. The salient point emerging from the above studies was the proposal that an A at −3 and G at +4 were important in the TIC, which was supported by the first two groups [40,41]. However, a more recent study by Agarwal *et al.*[43] demonstrates the functional significance of a TIC on the expression levels of a modified synthetic human α_1_-proteinase inhibitor (α_1_-P1) gene under 35S promoter in transgenic tomato plants. The researchers documented that there was negligible expression when the consensus bases at positions −3 and +4 were mutated, while in case of mutation only at position −3, there was a seven fold reduction in the expression levels.

The observations made in the present study, however, show that the influence of an optimum TIC (as present in synJ), seemed to be much more than what has been recorded in the literature reviewed. Almost 50% of the enhancing effect of the 5^′^UTR, synJ was due to the presence of the optimum plant TIC as the GUS activity obtained with 35S(synM) with mutated TIC was half than what was recorded in case of 35S(synJ) in transformed cotton callus. This trend was also observed in different tissues i.e. leaves, stems and roots of tobacco transgenic plants, which substantiated the results obtained in cotton.

In the present study, the complete TIC region, upstream to AUG has been mutated, without changing the +4 base. The higher level of drop in expression observed in the present study could be due to the multiple changes that have been carried out rather than changes in one or two bases. Although, not tested experimentally, if this is correct, then the whole sequence is important and not only the −3 and +4 bases, as proposed in the different studies. Our observations also show that the fold enhancement observed with synJ is not solely governed by an optimum TIC as mutating the upstream region (MsynJ) also leads to a drop similar to that visible by mutating the TIC (synM).

That a proper 5^′^UTR is important for realizing the full potential of a promoter is also highlighted in our observations. The 35S promoter has been suggested to be ~30 fold stronger than the nos promoter when the transcript levels were analyzed [32]. However, based on the GUS activity, the difference was observed to be ~110 fold. This variation in fold difference probably arises due to the presence of different 5^′^UTRs in the two constructs. When the synthetic 5^′^UTR of pRT100 was part of the *gus* transcript, no major difference in activity was observed [compare GUS activity of 35S(RT) and nos(RT) in Table 6 between the 35S and nos promoter. However, when nos or synJ is the 5^′^UTR of the *gus* transcript, 35S promoter shows ~10 fold more activity as studied in cotton callus. This shows that the outcome of promoter strength can be greatly influenced by the 5^′^UTR used, especially when the level of protein product is being used to report the strength of the promoter.

**Table 6:** Comparative GUS activity (pmolMU/min/μg protein) observed with 35S and nos promoter carrying constructs with different 5'UTRs in transformed cotton callus

**Construct**	**Expt No.**	**No. of callus tested**	**Gus activity (pmolMU/min/μg protein)**	
			**Range**	**Mean ± S.E.**
35S(nos)	I	27	67.5 - 1046.8	465.6 ± 47.1
	II	30	48.8 - 691.7	274.8 ± 30.8
	III	22	101.3 - 1077.0	580.6 ± 60.8
nos(nos)	I	20	22.0 - 100.1	54.4 ± 4.9
	II	22	35.5 - 98.4	64.6 ± 3.4
	III	22	28.8 – 114.8	70.4 ± 6.4
35S(synJ)	I	21	304.5 - 1916.9	989.8 ± 93.3
	II	29	222.5 - 2216.3	869.5 ± 99.1
	III	20	60.5 - 2389.0	1144.9 ± 254.0
nos(synJ)	I	16	37.9 - 314.6	131.2 ± 16.2
	II	22	54.6 - 364.7	177.2 ± 17.1
	III	27	73.7 - 577.5	287.7 ± 23.1
35S(RT)	I	25	2.7 - 12.7	7.8 ± 0.54
	II	26	3.3 – 11.3	7.03 ± 0.42
nos(RT)	I	23	4.9 – 15.6	9.33 ± 0.6
	II	25	5.9 – 19.4	9.4 ± 0.57

This led us to survey the literature where researchers have compared strengths of different promoters especially to the CaMV 35S. It turned out that in several reports [2,44-52] where promoter strengths were compared, the 5^′^UTR used were different between the different promoter constructs which could have greatly influenced their conclusions. These reports probably indicate the functionality of a URM (i.e. promoter and 5^′^UTR) rather than the promoter alone.

synJ as a 5^′^UTR of a transgene downstream to a promoter can be an alternative to using strong promoters or could also be used in conjunction with a strong promoter for achieving high levels of transgene expression. This strong influence of synJ was also observed in another study carried out in the laboratory [53], wherein the GUS activity observed with a truncated 35S promoter, containing only domain A of the wild-type 35S promoter was used. This study was not aimed at analyzing the influence of synJ but was carried out to study *trans*-inactivation of synthetic domain A of 35S promoter by “Tobacco 271 Locus”. The presence of synJ led to unexpectedly high levels of GUS activity even when *gus* was under the control of only the domain A of the 35S promoter. The GUS activity obtained with this truncated 35S promoter (carrying synJ as the 5^′^UTR) was similar to what others have recorded with the wild type 35S promoter [54,55]. 35S double enhancer promoter has been widely used to obtain high levels of gene expression as it yields ~10 fold higher expression in comparison to the wild type 35S promoter [22]. However, by employing synJ as a 5^′^UTR downstream to the 35S promoter, one can achieve even higher levels of protein product.

As the presence of synJ as a 5^′^UTR of transcripts of reporter genes like *gus* and *gfp* led to significant enhancement in the expression of transgenes in cotton callus as well as in different tissues of tobacco transgenic plants, we developed a set of cloning vectors pGEN01, pBGEN02, pBGEN02-*hpt* and pBGEN02-*ALS*^*dm*^ for cloning transgenes with synJ as a part of its 5^′^UTR which would be useful for those developing transgenic plants, needing high constitutive expression. The important features of these vectors are presented in Additional file 1: Figures S1, Additional file 2: Figure S2, Additional file 3: Figure S3 and Additional file 4: Figure S4 and restriction sites which could be used to clone a gene of interest are summarized in Additional file 5: Table S1, Additional file 6: Table S2, Additional file 7: Table S3 and Additional file 8: Table S4.

## Conclusions

The following conclusions can be drawn from this study:

1. The synthetic 5^′^UTR, synJ can efficiently enhance transgene expression under promoters like 35S and nos in dicotyledonous plants.

2. The enhancement obtained with synJ is similar to that of viral leaders like Ω and AMV. In the roots of tobacco transgenic plants, as well as in transformed cotton callus, synJ was much more efficient than both Ω and AMV. While in the stems, synJ is equivalent to AMV and much stronger than Ω. In the leaves, synJ was equivalent to Ω, while being weaker than that of the AMV leader sequence.

3. Although synJ carries a near consensus plant translation initiation context of 6 nt, the complete 28 nt is important for the observed enhancement.

4. The study also demonstrates the importance of 5^′^UTR in realizing the maximal potential of a promoter.

## Methods

### Development of constructs

The URMs, 35S(synJ), 35S(nos), 35S(MsynJ) and 35S(synM) were amplified for the development of respective constructs using the 35S promoter from pRT100 vector, as the template for the PCR reaction. A forward primer was designed from the distal end of the 35S promoter which had a *Hind*III and *Hinc*II site as an overhang. The reverse primer was designed from the proximal end of the promoter which carried the respective 5^′^UTR sequences synJ, nos, MsynJ, synM as an overhang along with an *Nco*I site at its 5^′^ end. The different URMs thus amplified were cloned upstream to the *gus* gene with a 35SpA available in pBlueScript vector as a *Hind*III-*Nco*I fragment. The sequences of the primers have been summarized in Additional file 9: Table S5. These expression constructs were then cloned into pPZP200N as a *Sac*I-*Sal*I fragment. To develop the vector 35S(pBI), the 35S promoter and a portion of the *gus* gene along with its 5^′^UTR (pBI) was excised from the binary vector pBI121 as a *Hind*III-*SnaB*I fragment. This 1.4 kb *Hind*III-*SnaB*I fragment of pBI121 was used to replace the *Hind*III-*SnaB*I fragment of the 35S(synJ):*gus*:pA cassette, present in pBSK. The complete expression cassette 35S(pBI):*gus*:35SpA, was cloned into the *Sac*I and *Sal*I sites in pPZP200N.

In order to develop *gfp* constructs, the gfp was cloned as a *Hinc*II-*Nco*I fragment in the pRT100 vector [21]. The 35S(RT)-*gfp*-35SpA cassette was then taken out as a *Hind*III fragment and cloned into the *Hind*III site of pPZP200N. The cloning of 35S(synJ)-*gfp*, was initiated by removing the upstream module 35S(RT) from the 35S(RT)-*gfp* construct in pRT100 and replacing it with the upstream module 35S(synJ), of the 35S(synJ)-*gus* construct as a *Hind*III-*Nco*I fragment. This cassette was further taken out from the pRT100 vector as a *Hind*III fragment and cloned into pPZP200N at the *Hind*III site.

The cloning of 35S(Ω) and 35S(AMV) was initiated in the ΔpBSK vector [devoid of the *Xho*I and *Kpn*I sites from the multiple cloning site of the pBSK+ vector (Stratagene)], carrying the 35S-*gus*-35SpA named as ΔpBSK 35SGUS was used for the cloning. The 5^′^UTR region of the CaMV promoter lies between the *Xho*I and *Nco*I sites of this construct. The AMV and the omega (Ω) leader sequences, to be cloned downstream to the 35S promoter in this construct, were created by annealing complementary oligos that were commercially synthesized (Additional file 9: Table S5). One of the oligos had *Xho*I site at its 5^′^ end as an overhang, while the other had the overhang of the *Nco*I site. The Ω and AMV 5^′^UTRs were cloned downstream to the 35S promoter in ΔpBSK 35SGUS as a *Xho*I-*Nco*I fragment. The 35S(Ω/AMV)-*gus*-35SpA cassettes were finally taken out and cloned into the binary vector pPZP200N as a *Sac*I-*Sal*I fragment.

In order to develop constructs nos(RT), nos(nos) and nos(synJ) carrying the promoter of the nopaline synthase gene, the nos promoter was amplified using a forward primer that was designed from the distal end of the promoter carrying a *Sa*cI site as an overhang and the reverse primer was designed from the proximal end of the promoter which carried the respective 5^′^UTR sequences nos, RT and synJ 5^′^UTRs as an overhang along with an *Nco*I site at its 5^′^ end. The sequences of the common forward primer and variant reverse primers are presented in Additional file 9: Table S5. Once the clones were assembled in the pBSK vector, the fidelity of the amplified products was checked by sequencing and finally the complete *gus* cassette was excised out and cloned into pPZP200N as a *Sac*I-*Sal*I fragment.

### Developing transformed cotton callus

Delinted seeds of cotton (*Gossypium hirsutum* L.cv. Coker 310 FR) were surface sterilized following Hemphill *et al.*[56] and germinated on half strength MS medium. Cotyledonary explants (approx. 1 cm^2^ area) from 7-day old seedlings were used for *A. tumefaciens* (strain GV3101 transformed with different binary vectors) infections [57] and explants were co-cultivated for 48 h at 22°C. The bacteriostatic and selective agent used was augmentin (250 mg/L) (Medreich Sterilab) and kanamycin (50 mg/L), respectively. The explants were placed on MS medium supplemented with 2,4-D (100 μg/L) and kinetin (500 μg/L) for inducing callus formation. 30 to 45 days old transformed callus were used for extraction of proteins to analyze *gus* or *gfp* expression levels.

### Development and maintenance of transgenics

Genetic transformation of tobacco (*Nicotiana tabacum* cv. Xanthi) was carried out using *A. tumefaciens* mediated transformation of leaf explants according to the protocol described by Svab *et al.* (1995) [58]. Several independent transgenic lines were developed with the constructs. Transgenic plants were maintained as shoot cultures in controlled environmental conditions (16 h day and 8 h night photoperiod, 28°C ± 2°C). For analyzing GUS activity in different tissues viz., in the leaf, root and stem, the tissues were harvested 40 to 45 days post subculture.

### Measurement of GUS activity and GFP levels

Total protein from different tissues of transgenic plants, seedlings and callus was extracted in GUS extraction buffer [59] followed by protein estimation by Bradford′s method [60]. Flourometric GUS assays using 4-methylumberrifyl-β-glucuronide (MUG) substrate were performed according to Jefferson [59]. The product released (MU) was estimated using a VersaFluor^TM^ Fluorometer (Bio-Rad) using excitation filter of 360 ± 20 nm and emission filter of 460 ± 5 nm. For a given set of samples 5 or 10 μg of protein was taken to carry out the reaction. In order to record the GUS activity in each sample, two reactions were carried out, one for 10 and another for 20 min. In the 20 min reaction, the value of F.U doubled in comparison to that of the 10 min reaction. GUS activity was recorded only for those samples, wherein the F.U/minute, were similar for the 10 and 20 minute reactions. In case of variation, the assay was repeated with different protein amounts depending upon the observed fluorescence. GUS activity was expressed as pmolMU/min/μg protein.

GFP activity was measured in 2 ml of 0.1 M Na_2_Co_3_ buffer with 500 μg protein of each extract and the readings were taken in fluorescence units (FU) on VersaFlour Flourometer (Bio-Rad) fitted with a 490 ± 5 nm excitation filter and a 510 ± 5 nm emission filter.

### RT-PCR (Reverse Transcription PCR)

For RT-PCR, total RNA was extracted with iRIS® total RNA isolation solutions (IHBT, Palampur, India) from the leaves of tobacco transgenic plants developed with 35S(RT) and 35S(synJ). The RNA isolated from the leaf tissue was treated with DNAse (DNAfree^TM^ kit, Applied Biosystems, USA) and before cDNA synthesis was carried out, DNAase treated RNA samples were tested for amplification with *ubiquitin* specific primers for 35 cycles. Only if the samples were negative for ubiquitin specific amplification (indicating absence of any DNA), the samples were taken forward for cDNA synthesis. The first strand cDNA was synthesized using oligo (dT)_16_ primer and MuLV reverse transcriptase enzyme (GeneAmp® RNA PCR, Applied Biosystems, USA) in a reaction volume of 100 μl. The reaction mix contained 1 μg of RNA, 10 μL reaction buffer, 20 μL of 500 μM dNTP, 22 μL of 25 mM MgCl_2_, 2 μL of RNAse inhibitor, 5 μL of 25 pmol/μL poly d(T) primer and 1 μL (50 U) of reverse transcriptase. This cDNA mix was used to carry out gene specific amplifications using two sets of primers. The primer set G(F): 5^′^-GCGCCATGGTACGTCCTGTAGAAACCCCAA-3^′^ and G(R): 5^′^-TGCCAGTTCAGTTCGTTGTTCA-3^′^ was designed to amplify a part of *gus* transcripts, while N(F): 5^′^-ATGGATTGCACGCAGGTTCT-3^′^ and N(R): 5^′^-TTCGCTTGGTGGTCGAATG-3^′^ amplified a region of the *nptII* transcript. The primer set U(F): 5^′^-GAAGGCATTCCACCTGACCAAC-3^′^ and U(R): 5^′^-CTTGACCTTCTTCTTCTTGTGCTTG-3^′^ was used to amplify a region of the ubiquitin transcript. The following reaction conditions were followed for *gus* amplification: a hot-start for 5 min at 94°C, variable cycles of 30 s at 94°C, 20 s at 60°C and 30 s at 72°C followed by an extension of 4 min at 72°C. In order to ensure that amplification was in the linear range, PCR was carried out for two different cycles e.g. 26 and 27 cycles in case of *gus* transcripts. The amplification of *ubiquitin* transcript was carried out as an internal control in all the samples to ensure that equal amount of cDNA was taken in each case.

The levels of transcripts (*gus*, *nptII* and *ubiquitin*) were quantified by measuring the intensity of the amplicon using AlphaImager program (HP). To normalize the variation in intensity between different gels due to different documentations, the intensity of the amplicon was divided by the intensity for the 500 bp band of the marker. A ratio of normalized *gus* transcript and normalized *nptII* (for 26 cycles) was used to analyze the transcript levels observed driven by different URMs.

## Abbreviations

AMV: Alfalfa mosaic virus; FMV: Figwort mosaic virus; MMV: Mirabilis mosaic virus; CaMV: Cauliflower mosaic virus; 5^′^UTR: 5^′^ untranslated region; URM: Upstream regulatory module; TMV: Tobacco mosaic virus.

## Competing interests

The authors’ declare that they have no competing interests.

## Authors' contributions

SK designed and performed all the experiments. PKB conceived this study and designed experiments. Both the authors wrote the manuscript and contributed equally in approving the final manuscript.

## Supplementary Material

Additional file 1 **Figure S1.** Map of the plasmid pGEN01 (JQ280537). The pGEN01 plasmid (3372 bp) carries an ampicillin resistance marker for selection in bacteria. The plasmid carries the P35S(synJ):35SpA cassette for attaining high level of gene expression. The desired gene can be cloned using the unique restriction enzyme sites present between the P35S(synJ) and 35SpolyA cassette. The complete cassette is flanked by *Sac*I sites present at the 2263 and 2963 positions in the plasmid, which can be used to take out the complete expression cassette from pGEN01 and cloned into a downstream binary vector. The restriction enzymes sites which can be used for cloning purposes have been marked. Click here for file

Additional file 2 **Figure S2.** Map of the binary vector pBGEN02 (JQ280534). The pBGEN02 plasmid (7882 bp) carries *aadA* gene conferring bacterial resistance to spectinomycin and streptomycin. The plasmid also carries the T-DNA left and right borders. As in the case of pGEN01, the plasmid carries the P35S(synJ):35SpA cassette for attaining high level of gene expression. The desired gene can be cloned using the unique restriction enzyme sites present between the 35S(synJ) and 35SpolyA cassette. The desired marker gene can be cloned at the *Hind*III or *Avr*II sites present within the *loxP*. The restriction enzymes sites which can be used for cloning purposes have been marked. Click here for file

Additional file 3 **Figure S3.** Map of the binary vector pBGEN02-*hpt* (JQ280535). The pGEN02-*hpt* plasmid (9621bp) carries *aadA* gene conferring bacterial resistance to spectinomycin and streptomycin. The plasmid carries an *hptII* selection marker cassette in between the *loxP* sites, which would facilitate the subsequent marker removal. As in the case of pBGEN02, the plasmid carries the P35S(synJ)-35SpA cassette for attaining high level of gene expression. The desired gene can be cloned using the unique restriction enzyme sites present between the 35S(synJ) and 35SpolyA cassette. The complete cassette is flanked by *Sac*I sites present at the 8530 and 9230 positions in the plasmid. The restriction enzymes sites which can be used for cloning purposes have been marked. Click here for file

Additional file 4 **Figure S4.** Map of the binary vector pBGEN02-*ALS*^*dm*^ (10549 bp) (JQ280536). pBGEN02-*ALS*^*dm*^ (10549 bp) carries *aadA* gene conferring bacterial resistance to spectinomycin and streptomycin. The plasmid carries an *ALS*^*dm*^ selection marker cassette in between the *loxP* sites, which would facilitate the subsequent marker removal. As in the case of pBGEN02, the plasmid carries the P35S(synJ)-35SpA cassette for attaining high levels of gene expression. The desired gene can be cloned using the unique restriction enzyme sites present between the 35S(synJ) and 35SpolyA cassette. The restriction enzymes sites which can be used for cloning purposes have been marked. Click here for file

Additional file 5 **Table S1.** List of important R.E. sites in pGEN01. Click here for file

Additional file 6 **Table S2.** List of important R.E sites in pGEN02. Click here for file

Additional file 7 **Table S3.** List of important R.E sites in pGEN02-*hpt.* Click here for file

Additional file 8 **Table S4.** List of important R.E sites in pGEN02-*ALS*^*dm*^*.* Click here for file

Additional file 9 **Table S5.** Sequences of the primers used in the cloning of constructs 35S(synJ), 35S(MsynJ), 35S(nos) and 35S(synM). Click here for file
