# Supplementary figures and images for: A 28 nt long synthetic 5′UTR (synJ) as an enhancer of transgene expression in dicotyledonous plants
Source: BMC Biotechnol. 2012 Nov 10;12:85. doi: 10.1186/1472-6750-12-85 (PMC3536603; doi:10.1186/1472-6750-12-85)

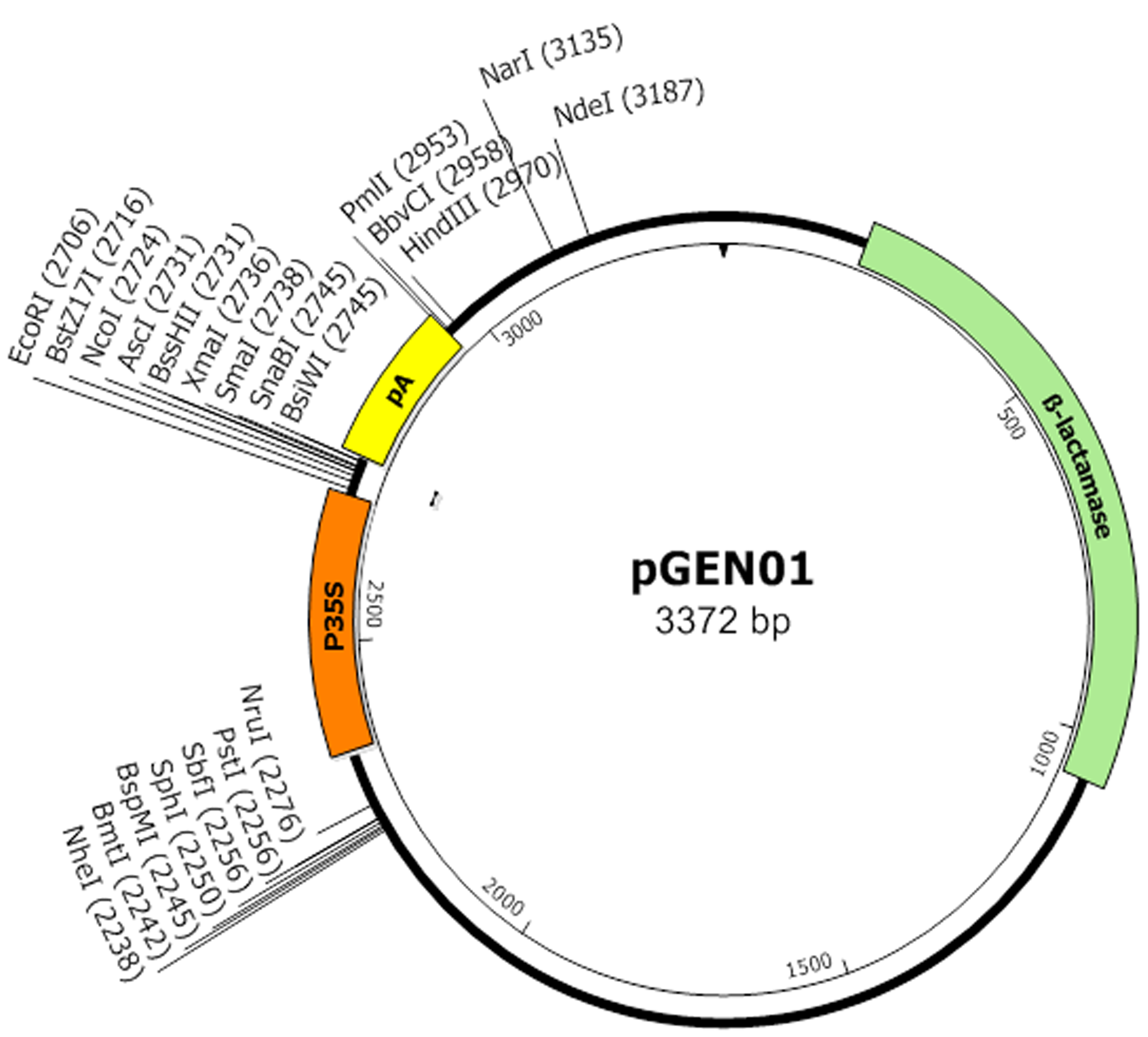

Supplement: Additional file 1 — Figure S1. Map of the plasmid pGEN01 (JQ280537). The pGEN01 plasmid (3372 bp) carries an ampicillin resistance marker for selection in bacteria. The plasmid carries the P35S(synJ):35SpA cassette for attaining high level of gene expression. The desired gene can be cloned using the unique restriction enzyme sites present between the P35S(synJ) and 35SpolyA cassette. The complete cassette is flanked by SacI sites present at the 2263 and 2963 positions in the plasmid, which can be used to take out the complete expression cassette from pGEN01 and cloned into a downstream binary vector. The restriction enzymes sites which can be used for cloning purposes have been marked. [file 1472-6750-12-85-S1.tiff]

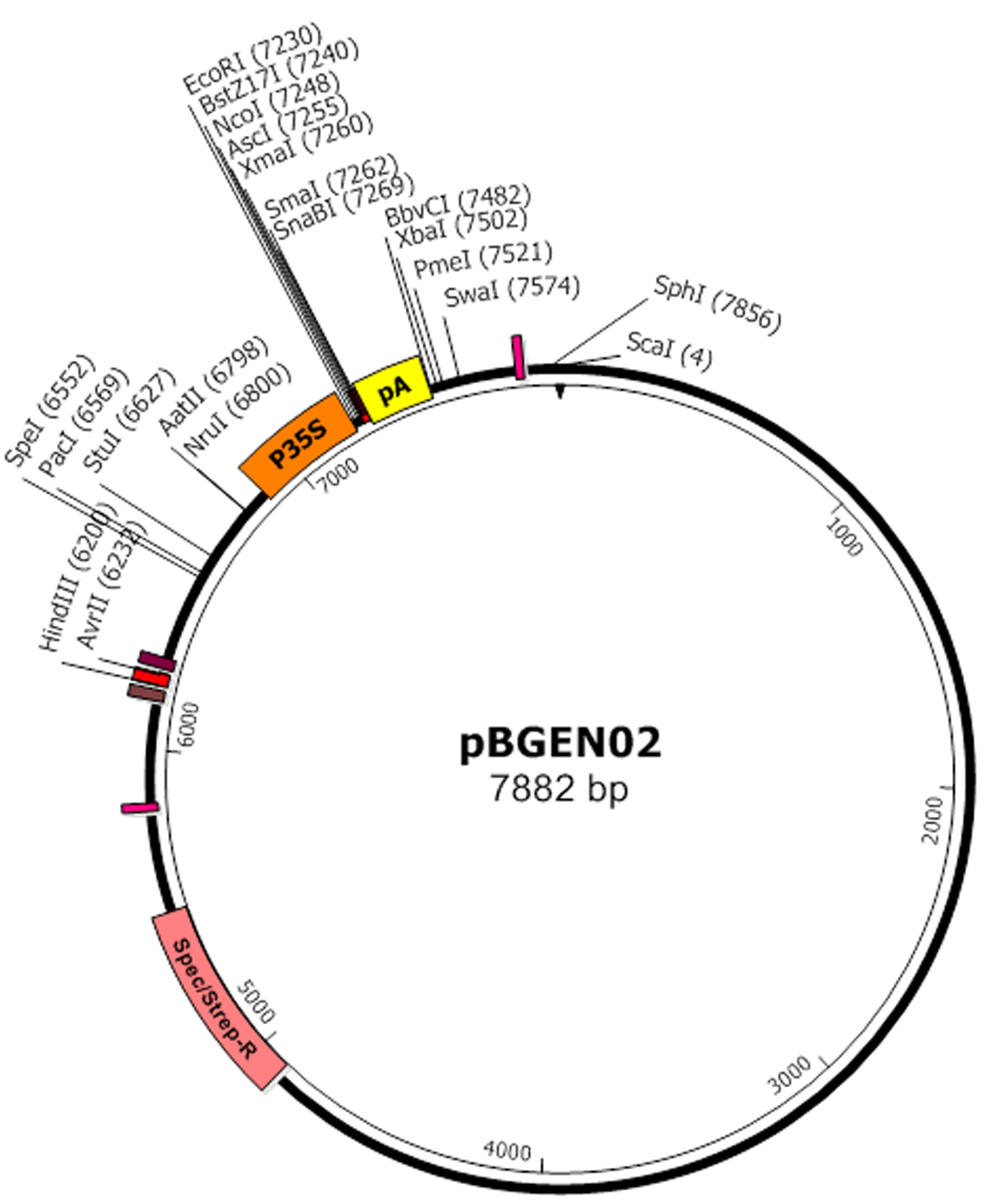

Supplement: Additional file 2 — Figure S2. Map of the binary vector pBGEN02 (JQ280534). The pBGEN02 plasmid (7882 bp) carries aadA gene conferring bacterial resistance to spectinomycin and streptomycin. The plasmid also carries the T-DNA left and right borders. As in the case of pGEN01, the plasmid carries the P35S(synJ):35SpA cassette for attaining high level of gene expression. The desired gene can be cloned using the unique restriction enzyme sites present between the 35S(synJ) and 35SpolyA cassette. The desired marker gene can be cloned at the HindIII or AvrII sites present within the loxP. The restriction enzymes sites which can be used for cloning purposes have been marked. [file 1472-6750-12-85-S2.tiff]

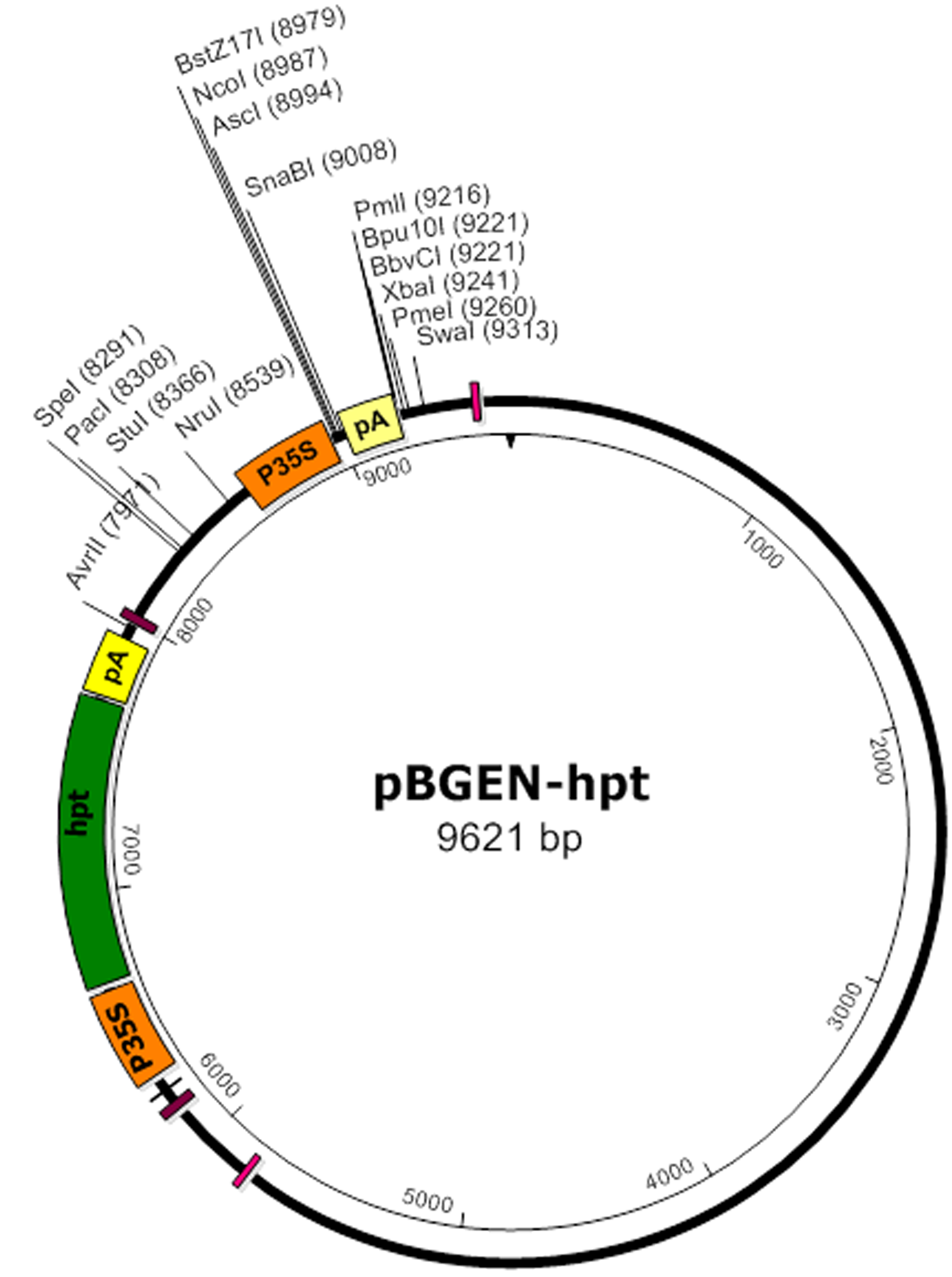

Supplement: Additional file 3 — Figure S3. Map of the binary vector pBGEN02-hpt (JQ280535). The pGEN02-hpt plasmid (9621bp) carries aadA gene conferring bacterial resistance to spectinomycin and streptomycin. The plasmid carries an hptII selection marker cassette in between the loxP sites, which would facilitate the subsequent marker removal. As in the case of pBGEN02, the plasmid carries the P35S(synJ)-35SpA cassette for attaining high level of gene expression. The desired gene can be cloned using the unique restriction enzyme sites present between the 35S(synJ) and 35SpolyA cassette. The complete cassette is flanked by SacI sites present at the 8530 and 9230 positions in the plasmid. The restriction enzymes sites which can be used for cloning purposes have been marked. [file 1472-6750-12-85-S3.tiff]

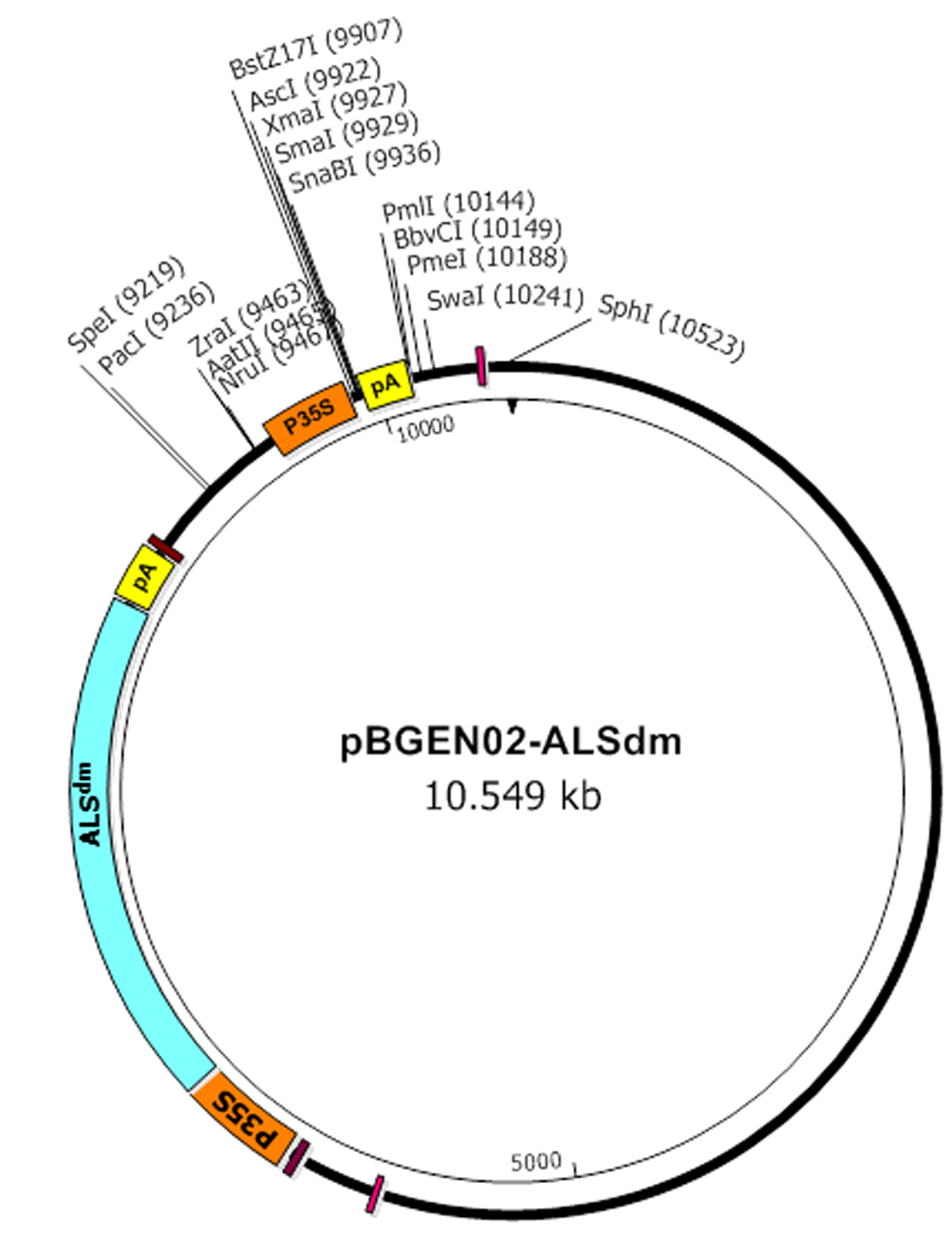

Supplement: Additional file 4 — Figure S4. Map of the binary vector pBGEN02-ALSdm (10549 bp) (JQ280536). pBGEN02-ALSdm (10549 bp) carries aadA gene conferring bacterial resistance to spectinomycin and streptomycin. The plasmid carries an ALSdm selection marker cassette in between the loxP sites, which would facilitate the subsequent marker removal. As in the case of pBGEN02, the plasmid carries the P35S(synJ)-35SpA cassette for attaining high levels of gene expression. The desired gene can be cloned using the unique restriction enzyme sites present between the 35S(synJ) and 35SpolyA cassette. The restriction enzymes sites which can be used for cloning purposes have been marked. [file 1472-6750-12-85-S4.tiff]
