# Supplementary material for: A 28 nt long synthetic 5′UTR (synJ) as an enhancer of transgene expression in dicotyledonous plants
Source: BMC Biotechnol. 2012 Nov 10;12:85. doi: 10.1186/1472-6750-12-85 (PMC3536603; doi:10.1186/1472-6750-12-85)
Supplement: Additional file 5 — Table S1. List of important R.E. sites in pGEN01. [file 1472-6750-12-85-S5.docx]

**Table S1:** List of important R.E. sites in pGEN01

| (A) Restriction Enzyme sites between 35S promoter and 35SpolyA signal in pGEN01 for cloning a gene of interest | | | (B) Restriction Enzyme sites for isolation of complete expression cassette | | |  |
| --- | --- | --- | --- | --- | --- | --- |
|  |  |  | 1. upstream to 35S promoter | | |  |
| **Enzyme** | **No. of sites** | **Location** | **Enzyme** | **No. of**  **sites** | **Location** |  |
| *EcoR*I | 1 | 2705 | *Nhe*I | 1 | 2237 |  |
| *BstZ*171 | 1 | 2715 | *Sph*I | 1 | 2250 |  |
| *Btg*I | 1 | 2723 | *Sbf*I | 1 | 2255 |  |
| *Sty*I | 1 | 2723 | *Pst*I | 1 | 2256 |  |
| *Nco*I | 1 | 2724 | *Hinc*II | 1 | 2260 |  |
| *Asc*I | 1 | 2730 | *Sac*I | 2 | 2267  2967 |  |
| *BssH*II | 1 | 2730 | *Nru*I | 1 | 2276 |  |
| *Ava*I | 1 | 2736 | (b) downstream to 35S polyA signal | | |  |
| *Xma*I | 1 | 2736 | *Pml*I 1 2952 | 1 | 2952 |  |
| *Sma*I | 1 | 2738 | *BbvC*I | 1 | 2957 |  |
| *Srf*I | 1 | 2737 | *Sac*I | 2 | 2267  2967 |  |
| *SnaB*I | 1 | 2744 | *Hind*III | 1 | 2970 |  |
|  |  |  | *Nar*I | 1 | 3134 |  |
|  |  |  | *Nde*I | 1 | 3187 |  |
