# Supplementary material for: A 28 nt long synthetic 5′UTR (synJ) as an enhancer of transgene expression in dicotyledonous plants
Source: BMC Biotechnol. 2012 Nov 10;12:85. doi: 10.1186/1472-6750-12-85 (PMC3536603; doi:10.1186/1472-6750-12-85)
Supplement: Additional file 6 — Table S2. List of important R.E sites in pGEN02. [file 1472-6750-12-85-S6.docx]

**Table S2:** List of important R.E sites in pGEN02

| (A) Restriction Enzyme sites between 35S promoter and 35SpolyA signal in pBGEN02 for cloning a gene of interest | | | | (C) Restriction Enzyme sites outside *loxP* in pBGEN02 (for cloning of marker gene) | | |  |
| --- | --- | --- | --- | --- | --- | --- | --- |
| **Enzyme** | **No. of sites** | | **Location** | **Enzyme** | **No. of**  **sites** | **Location** |  |
| *EcoR*I | 1 | | 7229 | *Ssp*I | 1 | 6539 |  |
| *BstZ*171 | 1 | | 7239 | *Spe*I | 1 | 6551 |  |
| *Nco*I | 1 | | 7247 | *Stu*I | 1 | 6626 |  |
| *Asc*I | 1 | | 7254 | *Aat*II | 1 | 6797 |  |
| *Sma*I | 1 | | 7261 | *Nru*I | 1 | 6799 |  |
| *Srf*I | 1 | | 7261 | 1. Restriction sites for cloning another transgene | | |  |
| *Xma*I | 1 | | 7259 | *Pml*I | 1 | 7476 |  |
| *SnaB*I | 1 | | 7268 | *BbvC*I | 1 | 7481 |  |
| (B) Restriction Enzyme sites between the *loxP* (for cloning of marker gene) | | | | *Xba*I | 1 | 7502 |  |
| *Hind*III | 1 | 6199 | | *Pme*I | 1 | 7520 |  |
| *Avr*II | 1 | 6231 | | *Swa*I | 1 | 7573 |  |
|  |  |  | | *Sph*I | 1 | 7855 |  |
