# Supplementary material for: A 28 nt long synthetic 5′UTR (synJ) as an enhancer of transgene expression in dicotyledonous plants
Source: BMC Biotechnol. 2012 Nov 10;12:85. doi: 10.1186/1472-6750-12-85 (PMC3536603; doi:10.1186/1472-6750-12-85)
Supplement: Additional file 7 — Table S3. List of important R.E sites in pGEN02-hpt. [file 1472-6750-12-85-S7.docx]

**Table S3:** List of important R.E sites in pGEN02-*hpt*

| (A) Restriction Enzyme sites between 35S promoter and 35SpolyA signal in pBGEN02-*hpt* for cloning a gene of interest | | | (D)Restriction Enzyme sites available in pBGEN02-*hpt*  for cloning another transgene | | |  |
| --- | --- | --- | --- | --- | --- | --- |
| **Enzyme** | **No. of sites** | **Location** | **Enzyme** | **No. of**  **sites** | **Location** |  |
| *BstZ*171 | 1 | 8978 | *Swa*I | 1 | 9312 |  |
| *Nco*I | 1 | 8986 | *Pml*I | 1 | 9215 |  |
| *SnaB*I | 1 | 9007 | *BbvC*I | 1 | 9220 |  |
| *Asc*I | 1 | 8993 | *Xba*I | 1 | 9240 |  |
| (B) Restriction Enzyme sites between the *loxP* in pBGEN02-*hpt* (for cloning of another marker gene) | | | *Pme*I | 1 | 9259 |  |
| *Avr*II | 1 | 7970 | *-* | - | - |  |
| C) Restriction Enzyme sites outside *loxP* in pBGEN02-*hpt* (for cloning of another marker gene) | | | *-* | - | - |  |
| *Ssp*I | 1 | 8278 | *-* | - | - |  |
| *Spe*I | 1 | 8290 | *-* | - | - |  |
| *Pac*I | 1 | 8307 | *-* | - | - |  |
| *Stu*I | 1 | 8365 | *-* | - | - |  |
| *Nru*I | 1 | 8538 | *-* | - | - |  |
