# Supplementary material for: A 28 nt long synthetic 5′UTR (synJ) as an enhancer of transgene expression in dicotyledonous plants
Source: BMC Biotechnol. 2012 Nov 10;12:85. doi: 10.1186/1472-6750-12-85 (PMC3536603; doi:10.1186/1472-6750-12-85)
Supplement: Additional file 8 — Table S4. List of important R.E sites in pGEN02-ALSdm. [file 1472-6750-12-85-S8.docx]

**Table S4:** List of important R.E sites in pGEN02-*ALS^dm^*

| (A) Restriction Enzyme sites between 35S promoter and 35SpolyA signal in pGEN02-*ALS^dm^* for cloning a gene of interest | | | (B) Restriction Enzyme sites outside *loxP* in pBGEN02-*als* (for cloning of another marker gene) | | |
| --- | --- | --- | --- | --- | --- |
| **Enzyme** | **No. of sites** | **Location** | **Enzyme** | **No. of sites** | **Location** |
| *BstZ*171 | 1 | 9906 | *Ssp*I | 1 | 9206 |
| *Asc*I | 1 | 9921 | *Spe*I | 1 | 9218 |
| *Xma*I | 1 | 9926 | *Aat*II | 1 | 9464 |
| *Sma*I | 1 | 9928 | *Nru*I | 1 | 9466 |
| *SnaB*I | 1 | 9935 | *-* | - | - |
| 1. Restriction Enzyme sites available in pBGEN02-*als*   for cloning another transgene | | | *-* | - | - |
|  |  |  | *-* | - | - |
| *Swa*I | 1 | 10240 | *-* | - | - |
| *Pml*I | 1 | 10143 | *-* | - | - |
| *BbvC*I | 1 | 10148 | *-* | - | - |
| *Pme*I | 1 | 10187 | *-* | - | - |
