# Supplementary material for: A 28 nt long synthetic 5′UTR (synJ) as an enhancer of transgene expression in dicotyledonous plants
Source: BMC Biotechnol. 2012 Nov 10;12:85. doi: 10.1186/1472-6750-12-85 (PMC3536603; doi:10.1186/1472-6750-12-85)
Supplement: Additional file 9 — Table S5. Sequences of the primers used in the cloning of constructs 35S(synJ), 35S(MsynJ), 35S(nos) and 35S(synM). [file 1472-6750-12-85-S9.docx]

**Table S5:** Sequences of the primers used in the cloning of constructs 35S(synJ), 35S(MsynJ), 35S(nos) and 35S(synM)

| 1. **Sequences of the primers used in the cloning of constructs**   **35S(synJ), 35S(MsynJ), 35S(nos) and 35S(synM)** | | |
| --- | --- | --- |
| Sequences of the reverse primers (5´ to 3´) | | Construct Name |
| 5´-TACCATggTTTAgTATACTAgAATTCCAgCgTgTCCTC  TCCAAATg-3´ | | 35S(synJ) |
| 5´-TACCATggTTTAgCTTCggATTGATAgCgCCTgTCCTC  TCCAAATg-3´ | | 35S(MsynJ) |
| 5´TCAACCATgggATTgAgAgTgAATATgAgACTCTgTCCTCTCAAATgAAATTgAACTT-3´ | | 35S(nos) |
| 5´-ggACCATggAAAAgTATACTAgAATTCCAgCgTgT-3´ | | 35S(synM) |
| Sequence of the forward primer | | |
| 5´-ATAAgCTTgTCAACATgAgACTTTTCAACAAAgggTAATATCggg-3´ | | |
| 1. **Sequences of the primers used in the cloning of constructs 35S(Ω) and 35S(AMV)** | | |
| 5´TCgAgTATTTTTACAACAATTACCAACAACAACAAACAACAAACAACATTACAATTACTATTTACAATTACAC 3´ | 35S(Ω) | |
| 5´CATggTgTAATTgTAAATAgTAATTgTAATgTTgTTTgTTgTTTgTTgTTgTTggTAATTgTTgTAAAAATAC-3´ |  |  |
| 5´TCgAgTTTTTATTTTTAATTTTCTTTCAAATACTTCCATCC 3´ | 35S(AMV) | |
| 5´CATgggATggAAgTATTTgAAAgAAAATTAAAAATAAAAAC 3´ |  |  |
| 1. **Sequences of the primers used in the development of constructs carrying nos promoter** | | |
| 5´ACTTATCCATGGGATTGAGAGTGAATATGAGAC  TCTAATTGGAT 3´ | nos(nos) | |
| 5´ACTTAACCATGGTTTAGTATACTAGAATTCCAG  CGTCTAATTGGATACCGAGGGGAATTTAT 3´ | nos(synJ) | |
| 5´TAGTCCATGGGCCCTCGAGCTAATTGGATACCGAGGGG 3´ | nos(RT) | |
| **Sequence of the forward primer** | | |
| 5´ATAAgCTTgTCAACATgAgACTTTTCAACAAAgggTAATATCggg 3´ | | |
